# Choose your own adventure: A perspective on career development

**DOI:** 10.1017/cts.2023.562

**Published:** 2023-06-26

**Authors:** Ansley Grimes Stanfill, Michelle Y. Martin

**Affiliations:** 1 Department of Acute and Tertiary Care, College of Nursing, University of Tennessee Health Science Center, Memphis, TN, USA; 2 Department of Preventive Medicine, College of Medicine, University of Tennessee Health Science Center, Memphis, TN, USA

**Keywords:** Career development, faculty development, retention, workforce, mentoring

## Introduction

Life in academia offers many opportunities, but the ideal career development goal looks different for everyone. Some faculty desire a research-intensive position, while others strive toward administrative roles, and still others prefer focusing on the educational mission of their institution. In the context of clinical and translational science, these paths all reflect exciting opportunities — from the challenges of discovery, to the joys of leadership, to the satisfaction of fostering the next generation—but in order to ensure continued success, faculty must align their talents and desires with the activities most suited for them. But this may be difficult, as some may lack role models or the confidence necessary to choose the career path that is the right fit. Recent trends, such as the “Great Resignation” and “quiet quitting,” suggest that many employees struggle with finding satisfaction in work; those in academia are not immune to these effects and may seek to find better work-life balance [1–3]. Finding personal meaning in one’s career path is central to finding long-term fulfillment and avoiding burnout.

Loosely inspired by the children’s book series, *Choose Your Own Adventure,* this perspective piece offers guidance on how to pinpoint your best career path in academia. In the book series, the reader encounters multiple scenarios, reviews their options, and selects what is most advantageous for them based on the circumstances. Two individuals reading the same *Choose Your Own Adventure* can read completely different stories based on the choices they make and the paths they choose to follow. This series is relevant as a metaphor for faculty career development, as it can provide a sense of self-efficacy and control as the master of one’s own journey by encouraging agency, open-mindedness, exploration, and diversity of experiences (especially for those with non-linear career paths in the clinical and translational sciences). Our goal in this paper is to discuss the process of choosing between options as they apply to a career path in academia, to describe common challenges, and to recommend solutions that may help readers pick the most fulfilling path for themselves. We hope to provide a framework that will help academicians choose their own adventure for their career path, which in turn will help maintain the health and well-being of our academic workforce in the clinical and translational sciences.

## Challenges and Solutions for Choosing Your Own Adventure

For some, it can be difficult to decide what sort of academic adventure they want to undertake. The reader of a *Choose Your Own Adventure* book may gather hints from the title, although the specifics of the journey will be different for each individual. In academia, most trainees start by following a traditional path. Usually, this means seeking a tenure-track position after completing postdoctoral training, with effort in this role being divided amongst teaching, service, and research/scholarship. For health science centers, clinical practice may also occupy a portion of the new faculty member’s time. Often, the talented faculty member advances during mid-career to administration or expanded service roles [4].

Whether following a tenure-track research path or deciding to choose a different academic adventure (e.g., administration, non-tenure track, or focusing more heavily on teaching or clinical practice), certain challenges may unfold at each new opportunity. As in the books, each prospect that is presented requires a decision about which path to follow. Ultimately, this process of decision-making may be reflective of the experiences we have had; our scientific discipline and mentorship experiences; as well as socialized expectations related to our cultural identity, gender orientation, and familial upbringing, among other factors. Each of these experiences and situations come with implicit and explicit biases that could influence the path we chose to take. Challenges to choosing your own career development adventure, however, are not insurmountable. Table 1 presents some solutions to these potential barriers.

**Table 1.** Potential challenges and solutions for choosing your own career development adventure

Potential challenge	Potential solution
Lack of knowledge of other career paths	Explore new activities, such as engaging in community-based research or working with an industry partner, that may open up novel career development paths. Find or organize interest groups and activities within your institution and professional organizations that highlight different academic career paths.
Lack of time to reflect on what the next phase of a career should be	Schedule a regular meeting time to reflect on your career journey and plan for the next stages.
Societal expectations of gender, age, discipline	Find mentors (even outside your own discipline) that are pushing back against expectations.
Overcommitment	Learn the value of saying no and being selective in which opportunities are taken

## A Mnemonic Reminder to Choose Your Own Career Adventure

When reading a *Choose Your Own Adventure* novel and facing the decision at the end of the chapter of which path you will choose, it can feel exciting and adventurous. In academia, however, it may be too overwhelming or scary to make that choice. However, therein lies an opportunity to take small steps using the ADVENTURE acronym we created to help academicians choose their career path. This acronym can also help scholars remain mindful and intentional about shaping their career.

**A**rticulate the contribution that you wish to make with your career.

**D**ecide to commit to a regular meeting time to reflect on your career journey and

plan for the next stages.

**V**erbalize the steps that are necessary to get to those next stages.

**E**xamine your calendar. Ensure your calendar activities align with your chosen academic adventure.

**N**egotiate with leadership to —

**T**ransform those activities that are not in alignment into something that contributes to the adventure you want to have or —

**U**nload those activities from your workload.

**R**eflect on ways to maintain accountability for the steps needed for your career development to move to the next stage (e.g., a friendly colleague that will hold you responsible).

**E**mbrace ownership of your adventure and explore other opportunities as your career develops.

## Conclusion

It is our hope that this paper will first assist faculty in identifying challenges to selecting their ideal career path. Next, we hope that our solutions will help faculty view their academic career path as a fun adventure and allow them to overcome these challenges. The mnemonic should also serve as a reminder to continuously seek out the career path that is most fulfilling.
